# Gross Intrahepatic Mass Formation Predicts the Primary Site of Perihilar Cholangiocarcinoma Based on Molecular Pathologic Studies

**DOI:** 10.1002/jhbp.70077

**Published:** 2026-02-01

**Authors:** Yuki Masuda, Naoto Kubota, Ryo Takemura, Yasuhito Arai, Yuta Abe, Osamu Itano, Minoru Esaki, Tatsuhiro Shibata, Yuko Kitagawa, Hidenori Ojima

**Affiliations:** ^1^ Department of Surgery Keio University School of Medicine Shinjuku‐ku Japan; ^2^ Department of Pathology Keio University School of Medicine Shinjuku‐ku Japan; ^3^ Research Institute, Division of Molecular Pathology Tochigi Cancer Center Utsunomiya Japan; ^4^ Division of Cancer Genomics National Cancer Center Research Institute Chuo‐ku Japan; ^5^ Biostatistics Unit, Clinical and Translational Research Centre Keio University Hospital Shinjuku‐ku Japan; ^6^ Department of Hepato‐Biliary‐Pancreatic and Gastrointestinal Surgery International University of Health and Welfare School of Medicine Narita Japan; ^7^ Department of Hepatobiliary and Pancreatic Surgery National Cancer Center Hospital Chuo‐ku Japan

**Keywords:** alpha 1‐antitrypsin, biomarkers, cholangiocarcinoma, claudins, mesothelin

## Abstract

**Background/Purpose:**

Intrahepatic cholangiocarcinoma (iCCA) and extrahepatic cholangiocarcinoma (eCCA) are clinically and genetically distinct. However, the classification of perihilar cholangiocarcinoma (phCCA) with an intrahepatic tumor mass remains unclear. This study aimed to position phCCA near the hilar plate (hCCA) within an extrahepatic–intrahepatic framework using pathological and molecular analyses.

**Methods:**

Among 357 resected invasive CCAs, 100 hCCAs were histologically classified as either hCCA with (hCCA‐M) or hCCA without (hCCA‐NM) a grossly evident intrahepatic mass. Transcriptomic comparison of 9 typical eCCAs and 39 mass‐forming iCCAs identified three contextual markers, which were examined by immunohistochemistry in 309 additional cases.

**Results:**

Among 100 hCCAs, 85 were hCCA‐NM and 15 hCCA‐M. Claudin 18 (CLDN18) and mesothelin (MSLN) were identified as extrahepatic contextual markers, and serpin family A member 1 (SERPINA1) as an intrahepatic contextual marker. SERPINA1 was more highly expressed in hCCA‐M than in hCCA‐NM, regardless of microscopic liver parenchymal invasion, whereas CLDN18 and MSLN were similarly expressed in both. Cluster analysis revealed that hCCA‐NM clustered with eCCA, whereas hCCA‐M clustered with iCCA.

**Conclusions:**

Gross intrahepatic mass formation indicates an intrahepatic contextual profile and provides a useful criterion for subclassifying hCCA. This contextual framework shows that hCCA‐M and hCCA‐NM represent biologically distinct tumor groups.

## Graphical abstract

Masuda and colleagues showed that perihilar cholangiocarcinoma near the hilar plate is biologically heterogeneous. Tumors without a gross intrahepatic mass resembled extrahepatic cholangiocarcinoma, whereas those with a gross intrahepatic mass demonstrated an intrahepatic molecular profile, indicating distinct biological subgroups and providing insight into the primary site of origin.

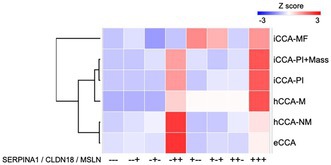

## 1 Introduction

Cholangiocarcinoma (CCA) is one of the most refractory cancers and is on the increase worldwide [1]; moreover, there has been little improvement in prognosis [2]. Surgical resection remains the only radical treatment, but many cases are advanced and unresectable at the time of clinical diagnosis [3]. Furthermore, a high rate of recurrence occurs even after surgical resection [4]. Chemotherapy is indicated for unresectable and postoperative recurrence cases, but current chemotherapy drugs for CCAs, such as gemcitabine and cisplatin, are not sufficiently effective. Recently, molecularly targeted drugs against specific genetic abnormalities have been introduced for CCA treatment [5], and the distribution of these genetic abnormalities was diverse among the primary sites of CCAs from large‐scale genome analysis [6]. For example, inhibitors of isocitrate dehydrogenase type 1 (IDH1) are effective for CCAs with a mutated form of IDH1, which is frequently found in intrahepatic CCA (iCCA) but not in extrahepatic CCA (eCCA). Moreover, different biological behaviors of CCAs depending on the primary site have been reported [7, 8], and the causes and the risk factors of CCAs are different by primary site [9]. Consequently, an accurate diagnosis of the primary site of CCA is important for successful treatment.

However, the diagnosis of CCA located near the hilar plate, especially with intrahepatic tumor mass, is difficult because the hilar region is the border area of two embryologically distinct organs: the extrahepatic and intrahepatic bile ducts [10, 11]. No general clinicopathologic evaluation criteria have been established to differentiate perihilar invasion of iCCA from liver parenchymal invasion of eCCA. Therefore, the World Health Organization (WHO)/Union for International Cancer Control/American Joint Committee on Cancer currently define perihilar cholangiocarcinoma (phCCA) as a CCA mainly located in the hilar area; this definition can include tumors with unclear primary sites that could have arisen from either the extrahepatic or intrahepatic bile duct [12]. Considering the genetic and biological differences that depend on the primary site, the diagnosis of phCCAs with an unclear primary site is a major problem that must be resolved to apply appropriate medication and improve prognosis.

The aim of this study was to identify the primary site of phCCA near the hilar plate (hCCA) using molecular pathological studies in addition to the detailed histological evaluation criteria of CCA.

## 2 Methods

### 2.1 Patients

Five hundred patients who underwent resection of CCA at the National Cancer Center Hospital (Tokyo, Japan) between 1990 and 2013 were included in this study. Carcinoma in situ, intraductal papillary neoplasms of the bile duct, and cases for which the primary site was difficult to identify due to widespread involvement throughout the entire bile duct were excluded. As a result, 357 cases of intrahepatic, perihilar, and distal extrahepatic bile duct carcinomas were selected. This study was performed in accordance with the Declaration of Helsinki, subsequent to approval from the institutional review board of the National Cancer Center (#2007‐022). All patients gave written informed consent.

### 2.2 Definition of the Primary Site

The determination of the primary site was based on macroscopic evaluation initially and confirmed by histopathological evaluation as follows [7]. The gross findings of the resected specimen, including the cut surfaces, were recorded in detail along with comparisons to computed tomography imaging findings such as bile duct stricture. After fixation of the specimens in 10% formalin, the liver parenchyma was axially cut into 5‐mm‐thick slices; branches of the bile duct and vasculature were annotated in all tissue slices. Additionally, serial sagittal sections 3–5 mm thick were taken around the hilar region when necessary to assess the precise tumor area. Identification of all vascular and portal areas was confirmed with imaging findings. In the distal bile duct, serial sections were taken perpendicular to the longitudinal axis of the bile duct.

**FIGURE 1:**
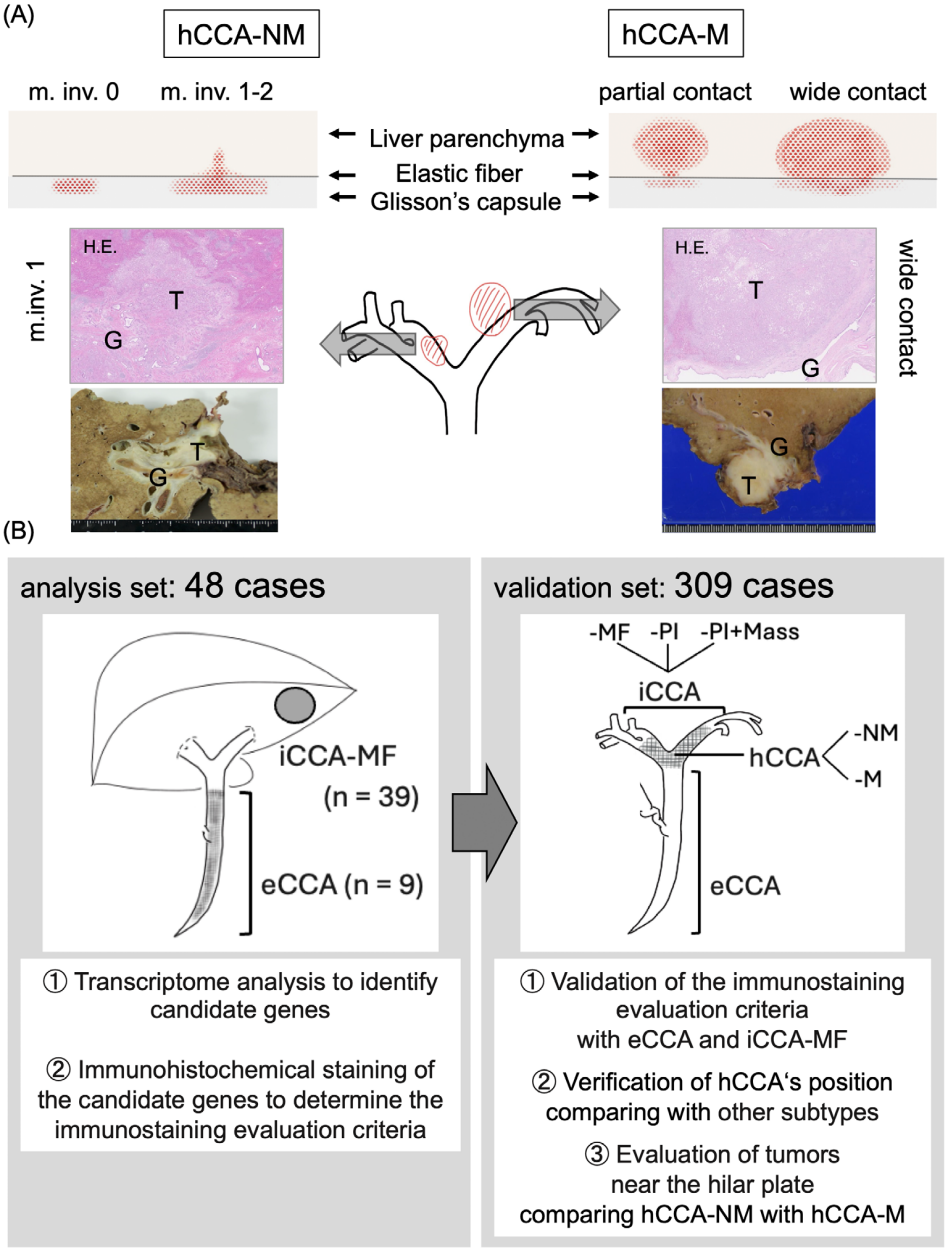
Study design and histopathological criteria for subclassifying hCCA. (A) Diagnostic criteria for differentiation between perihilar cholangiocarcinoma near the hilar plate (hCCA) without a grossly evident intrahepatic mass (hCCA‐NM) and hCCA with a grossly evident intrahepatic mass (hCCA‐M). Microscopic liver parenchymal invasion of hCCA‐NM is typically wedge‐shaped with indistinct or irregular borders. hCCA‐M typically has a round mass with clear and regular borders in the liver parenchyma. The liver parenchymal mass of hCCA‐M is often in partial contact or in wide contact with the Glisson's capsule and is invading the Glisson's capsule. m.inv.0, no liver parenchymal invasion; m.inv.1, liver parenchymal invasion of less than 5 mm; m.inv.2, liver parenchymal invasion of 5–20 mm. H.E., hematoxylin–eosin stain; T, tumor; G, Glisson's capsule. (B) Analysis and validation sets and procedures followed in this study. In the analysis set, extrahepatic cholangiocarcinoma (eCCA) and mass‐forming intrahepatic cholangiocarcinoma (iCCA‐MF) with clear distribution and no hilar plate invasion were compared. In the validation set, 309 cases, excluding the analysis set cases, were classified by subtype. MF, mass‐forming type; PI, periductal infiltrating type; PI+Mass, periductal infiltrating plus mass‐forming type.

iCCA was defined as a tumor with its primary in the second‐order or more peripheral branches of the bile duct. iCCAs were pathologically classified into three macroscopic types according to the WHO Classification of Tumors: Digestive System, 5th ed. (2019), with reference to the General Rules for the Clinical and Pathological Study of Primary Liver Cancer (Liver Cancer Study Group of Japan) [13], i.e., mass‐forming type (MF) with nodules in the liver parenchyma, periductal infiltrating type (PI) extending along the portal tracts, and periductal infiltrating plus mass‐forming type (PI+Mass). eCCA was defined as a tumor originating distal to the upper bile ducts. hCCA (phCCA near the hilar plate) was defined as a CCA being located between iCCA and eCCA. We subclassified hCCA into the following two categories: (i) hCCA without a grossly evident intrahepatic mass (hCCA‐NM) and (ii) hCCA with a grossly evident intrahepatic mass (hCCA‐M). If, under microscopic assessment, hCCA‐NM was found to have liver parenchymal invasion, we further classified hCCA‐NM based on the depth of liver parenchymal invasion as microscopic liver parenchymal invasion (m.inv.) (for details, see Figure 1A). m.inv. is characterized by a small amount of invasion (< 5 mm) into liver parenchyma, or by liver parenchymal invasion without a grossly evident intrahepatic mass, i.e., the presence of minor wedge‐shaped invasion (~5–20 mm). As in our previous report on the histological evaluation of CCA [7], Elastica van Gieson staining was used in addition to hematoxylin–eosin staining to evaluate the relationship of the tumor to the hilar plate/intrahepatic Glisson's capsule to accurately diagnose the tumor location.

### 2.3 Selection of the Analysis and Validation Sets

The analysis set was selected from typical eCCA and iCCA‐MF cases resected between 2003 and 2013 with clear tumor distribution and without hilar plate invasion. Nine cases of eCCA and 39 cases of iCCA‐MF (all small duct type) were selected for the analysis set; for all these cases, both frozen and formalin‐fixed paraffin‐embedded (FFPE) samples were available. An additional 309 cases having FFPE specimens were selected as the validation set.

### 2.4 Microarray Gene Expression Analysis and Identification of Candidate Markers

Since 2003, fresh frozen specimens have been taken from the main lesion of the tumor and stored at −80°C. Total RNA was extracted from whole frozen specimens using an RNeasy Mini Kit (Qiagen, Hilden, Germany) according to the manufacturer's instructions. Cases with RIN > 7.5 were selected. An Agilent One Color Human 60K Array V1 (SurePrintG3#28004) was used, and analysis was performed using R‐Limma, as described previously [14]. Because frozen iCCA‐MF specimens often included non‐neoplastic liver parenchyma while eCCA specimens did not, a liver‐reference adjustment was first performed using non‐neoplastic liver profiles from nine metastatic liver resections (histologically tumor‐free) to estimate and subtract the liver‐derived signal. Normalization and linear modeling (R‐Limma) were then applied to the eCCA–iCCA‐MF comparison in the analysis set. Differentially expressed genes [false discovery rate (FDR) < 0.02] between eCCA and iCCA‐MF were identified. Fold change values (FC > 6) were applied to identify reliable marker genes. For the contextual marker nomination process, we applied four pre‐specified, practical criteria: (i) feasibility and robustness for FFPE immunohistochemistry (guided by the Human Protein Atlas (https://www.proteinatlas.org) and prior reports), (ii) interpretability for positioning tumors along an extrahepatic–intrahepatic context, (iii) small pilot IHC checks, where feasible, to confirm interpretable staining, and (iv) prioritization of molecules previously reported in the literature for extrahepatic and intrahepatic cholangiocarcinoma, to avoid selecting markers that merely showed statistical separation without biological interpretability. Available and stable antibodies were prioritized using the Human Protein Atlas; because eCCA is not listed there, pancreas (embryologically and pathologically related to eCCA) [10, 11, 15] was used as a surrogate reference.

### 2.5 Immunohistochemical Staining

For immunohistochemical analysis, sections with a sufficient tumor burden that reflected the overall picture and with predominant differentiation and histology were selected as representative sections by an experienced pathologist (HO). Formalin‐fixed, paraffin‐embedded, 4‐μm‐thick serial tissue sections were placed on silane‐coated slides for immunohistochemical analysis. Sections were deparaffinized in xylene, rehydrated in 50%–100% diluted ethanol, and then immersed in 0.3% hydrogen peroxide in absolute methanol for 20 min to inhibit endogenous peroxidase activity. The reagents and conditions used in immunohistochemical staining are indicated in Data S1. All immunohistochemical evaluations were performed by two of the authors (Y.M. and H.O.). If the initial evaluation yielded different results, consensus was reached after repeat examination. Figure 1B shows the overall design of this study.

### 2.6 Statistical Analysis

The Mann–Whitney *U* test was used for gene expression analysis, and Fisher's exact test was used to evaluate the immunohistochemical staining results. A *p* value of less than 0.05 between two groups was considered statistically significant. Multiplicity was adjusted using the Bonferroni method. Heat maps were generated by hierarchical clustering with Pearson correlation using cmap/morpheus.R (https://github.com/cmap/morpheus.R).

## 3 Results

### 3.1 Classification of CCA and Patient Characteristics

Based on our classification criteria, the validation cohort was composed of 100 eCCA, 85 hCCA‐NM, 15 hCCA‐M, and 109 iCCA tumors. For most tumors, especially for those with an intrahepatic tumor mass, the histological tumor area was similar to the macroscopic appearance. Three cases initially recorded as lacking a grossly evident intrahepatic mass were found on microscopic review to have contiguous liver parenchymal invasion exceeding 20 mm. As part of our standard quality‐control process, the relevant gross photographs were rechecked, and all three showed a gross intrahepatic mass, leading to their reclassification as hCCA‐M. Table 1 summarizes the details of our cohorts.

**TABLE 1:** Patient characteristics of the analysis and validation sets.

	Analysis set		Validation set						
	eCCA	iCCA‐MF	eCCA	hCCA‐NM	hCCA‐M	iCCA			
						Total	iCCA‐PI	iCCA‐PI+Mass	iCCA‐MF
Number of patients	9	39	100	85	15	109	14	36	59
Age, years [range]	71 [61–80]	69 [26–81]	67 [43–83]	62 [41–83]	66 [41–73]	65 [35–89]	67 [50–89]	67 [35–80]	63 [44–84]
Sex (male/female)	7/2	11/28	84/16	60/25	9/6	67/42	10/4	20/16	37/22
Tumor size, cm (median [range])	6.5 [4–8]	4.8 [1.9–13]	4.5 [0.3–10]	3.5 [1–15]	4 [1.8–6.5]	4.5 [1.4–15]	3.7 [1.5–8.5]	3.5 [1.4–11]	5.4 [2–15]
Differentiation (well/moderate/poor)	2/7/0	7/29/2	16/63/21	25/55/5	3/11/1	19/87/2	4/10/0	4/31/0	11/46/2
Venous invasion (%)	9 (100)	31 (79)	96 (96)	80 (94)	15 (100)	95 (87)	14 (100)	34 (94)	47 (80)
Lymphatic invasion (%)	9 (100)	28 (72)	97 (97)	79 (93)	15 (100)	93 (85)	14 (100)	36 (100)	43 (73)

Abbreviations: eCCA, extrahepatic cholangiocarcinoma; hCCA, perihilar cholangiocarcinoma near the hilar plate; hCCA‐M, hCCA with a grossly evident intrahepatic mass; hCCA‐NM, hCCA without a grossly evident intrahepatic mass; iCCA, intrahepatic cholangiocarcinoma; MF, mass‐forming type; PI, periductal infiltrating type; PI+Mass, periductal infiltrating plus mass‐forming type.

### 3.2 Gene Expression in the Analysis Set and Selection of Candidate Marker Genes With Differential Expression in eCCA and iCCA‐MF

**FIGURE 2:**
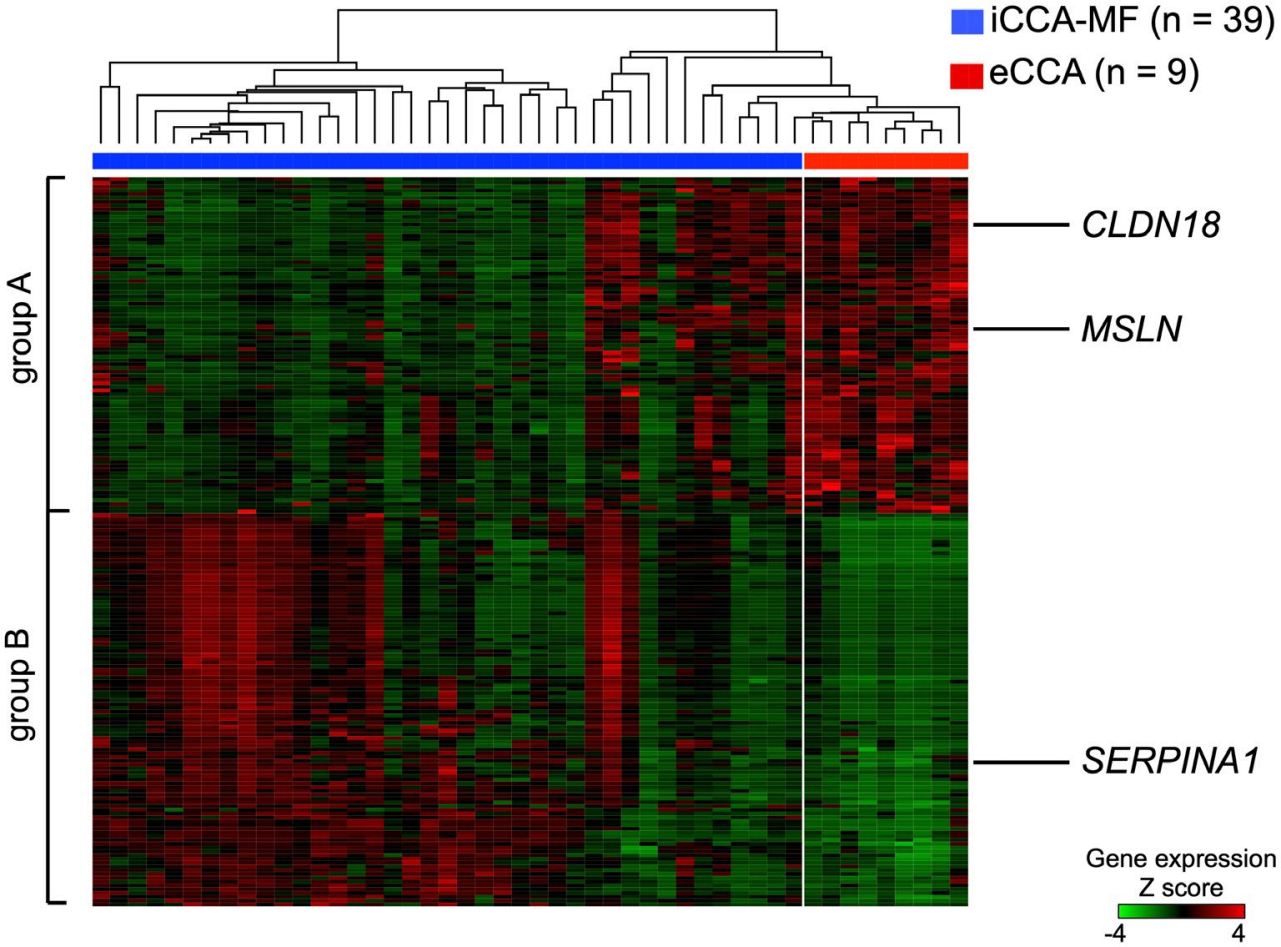
Heat map of gene expression comparing eCCA with iCCA‐MF in the analysis set. The heat map was created for 193 genes with FDR < 0.02 and FC > 6, ordered by FC value. SERPINA1 was highly expressed in iCCA‐MF, whereas CLDN18 and MSLN were highly expressed in eCCA. Group A represents genes that were highly expressed in almost all cases of eCCA. Group B indicates genes with low expression in eCCA and high expression in most cases of iCCA‐MF. SERPINA1, serpin family A member 1; CLDN18, claudin 18; MSLN, mesothelin.

By comparing eCCA and iCCA‐MF tumors in the analysis set, 1462 genes were extracted with FDR < 0.02. In total, 193 genes with FC > 6 were identified and sorted in FC order (Figure 2). From these 193 candidates, a pragmatic, fit‐for‐purpose nomination process was applied using four pre‐specified criteria (see Methods). Claudin 18 (CLDN18) (rank 19) and mesothelin (MSLN) (rank 159) were selected as extrahepatic candidate contextual marker genes, and serpin family A member 1 (SERPINA1) (rank 76) was selected as an intrahepatic candidate contextual marker gene.

### 3.3 Immunohistochemical Expression of Three Candidate Marker Proteins in the Analysis Set, Cut‐Off Values, and Gene Expression Levels

**FIGURE 3:**
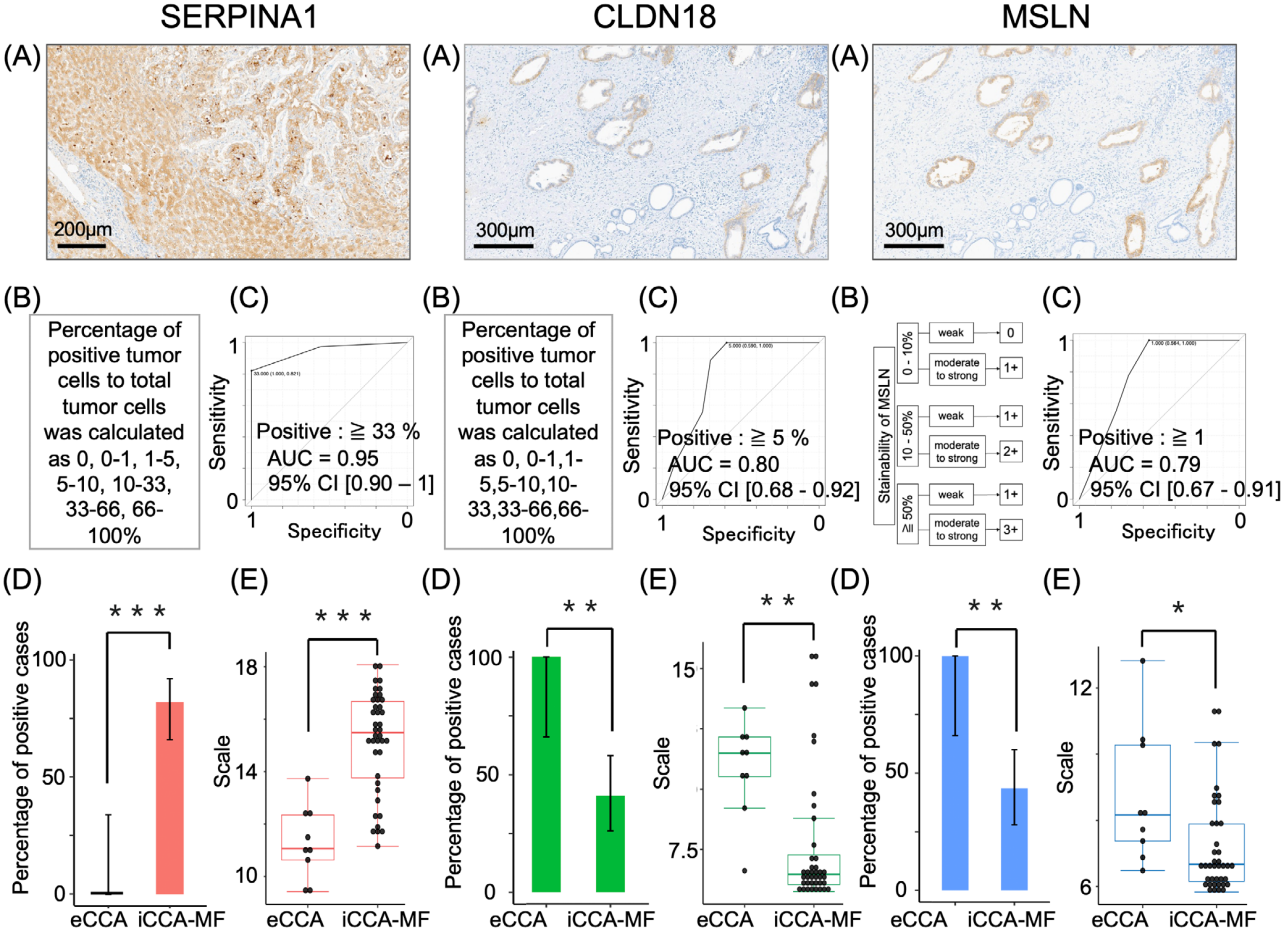
(A) Representative immunostaining patterns. SERPINA1: iCCA‐MF; CLDN18: eCCA; MSLN: eCCA. SERPINA1 occasionally showed focally stronger staining at the liver parenchymal invasive front, with a slight rise in the percentage of positive tumor cells; however, in our cohort, this was insufficient to exceed the ≥ 33% cutoff and did not affect the positivity status. The distributions of CLDN18‐ and MSLN‐positive cancer cells were relatively homogeneous within the tumor, and the degree of expression was rarely affected by liver parenchymal or stromal invasion. (B) Evaluation criteria for immunostaining. (C) Cut‐off values were determined based on the ROC curves. Tumors with more than 33% of SERPINA1, more than 5% of CLDN18, or more than 1+ of MSLN were designated as positive. (D) Percentage of positive cases for eCCA and iCCA‐MF. These percentages were significantly different between eCCA and iCCA‐MF for all three markers. (E) The pattern of gene expression between eCCA and iCCA‐MF. Gene expression levels were similar to the levels of the immunohistochemical staining between eCCA and iCCA‐MF. Error bars indicate 95% confidence intervals. *p < 0.05, **p < 0.01, ***p < 0.001. Red bars, SERPINA1; green bars, CLDN18; blue bars, MSLN.

Criteria for positive immunohistochemical staining of the proteins encoded by the three candidate contextual marker genes were developed using procedures modified from previous reports [16, 17, 18, 19, 20, 21, 22, 23]. Representative staining patterns of the three contextual markers are shown in Figure 3A. For SERPINA1 and CLDN18, the positivity rates were categorized into seven groups (0%, 0%–1%, 1%–5%, 5%–10%, 10%–33%, 33%–66%, 66%–100%), and for MSLN, a scoring system was applied (see Figure 3B and Data S1). Cut‐off values for the extent of immunohistochemical staining for the three candidate marker proteins were calculated using ROC curves to define positive cases in this study. The optimum cut‐off values were SERPINA1 ≥ 33%, CLDN18 ≥ 5%, MSLN ≥ 1+ (see Figure 3C). Protein expression by immunostaining and gene expression were consistent for each marker candidate (Figure 3D,E).

### 3.4 Immunohistochemical Expression Analysis of Three Contextual Marker Proteins in the Validation Set

**FIGURE 4:**
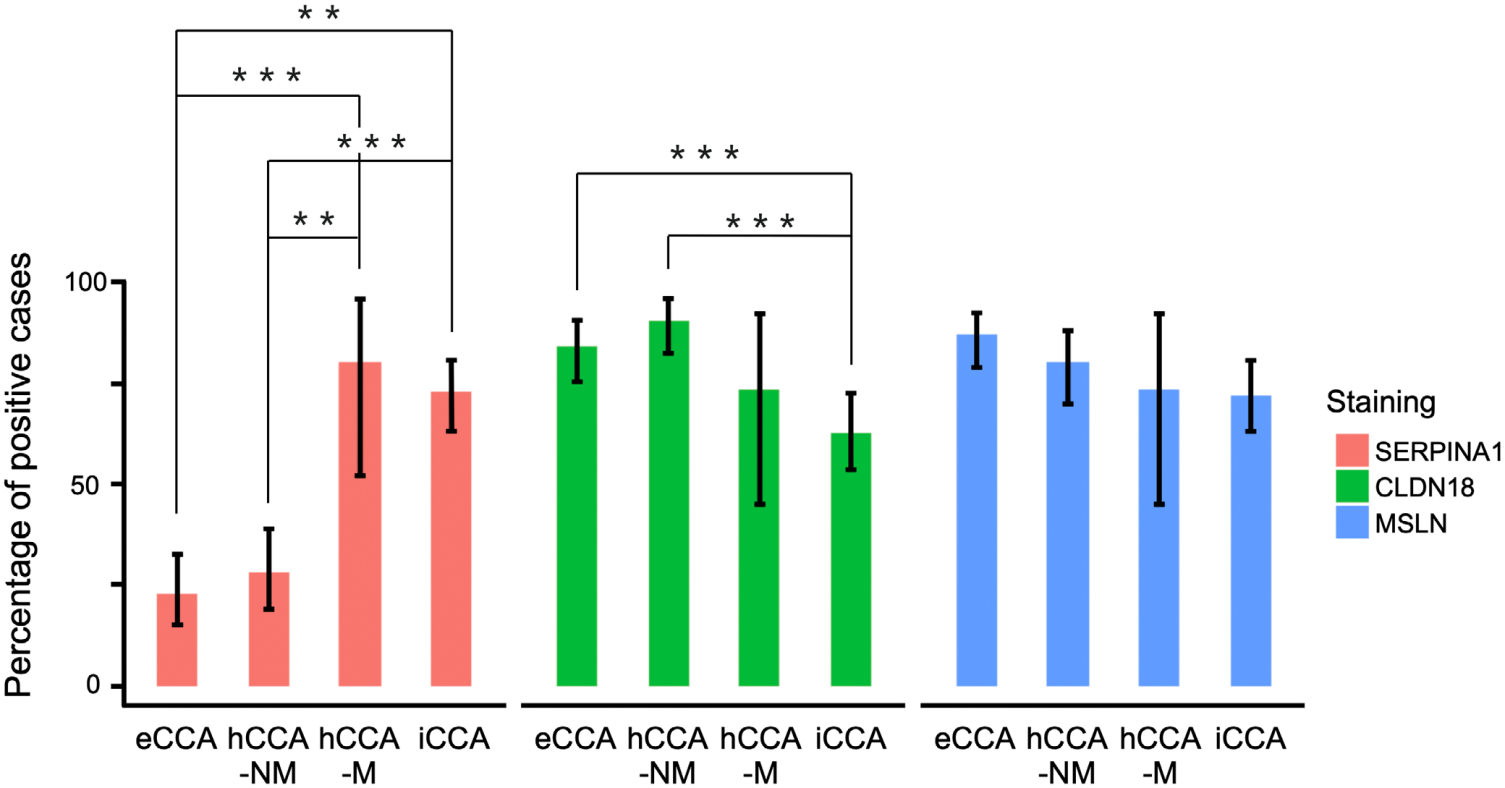
Percentages of positive cases of eCCA, hCCA‐NM, hCCA‐M, and iCCA in the validation set calculated based on the staining cut‐off values of the three markers. hCCA‐NM and hCCA‐M were significantly different in terms of SERPINA1 positivity. eCCA and hCCA‐NM had similar levels of positivity for all three stains. Positivity levels of hCCA‐M and iCCA were also similar for all three stains. Error bars indicate 95% confidence intervals. **adjusted *p* < 0.01, ***adjusted *p* < 0.001.

The classification criteria of immunostaining developed in the analysis set were applied to the validation set (Figure 4). For all three contextual markers, there were significant differences in the percentage of positive cases between eCCA and iCCA‐MF (Data S2): SERPINA1, 23% vs. 81% (*p <* 0.001); CLDN18, 84% vs. 37% (*p <* 0.001); MSLN 87% vs. 61% (*p <* 0.001). There was also a significant difference between hCCA‐NM and hCCA‐M for SERPINA1 (28% vs. 80%, adjusted *p <* 0.01), but no difference between these two subtypes for CLDN18 (adjusted *p =* 0.47) or MSLN (adjusted *p =* 1.00). There was a significant difference between hCCA‐NM and iCCA for SERPINA1 (28% vs. 72%, adjusted *p <* 0.001) and CLDN18 (91% vs. 63%, adjusted *p <* 0.001) but not for MSLN (adjusted *p =* 1.00). There were no significant differences between eCCA and hCCA‐NM (adjusted *p =* 1.00 for all three markers). Representative immunohistochemical panels for hCCA‐M and hCCA‐NM are provided in Data S3.

### 3.5 Cluster Analysis of Tumor Subtypes Based on Expression Patterns of the Three Markers in the Validation Set

**FIGURE 5:**
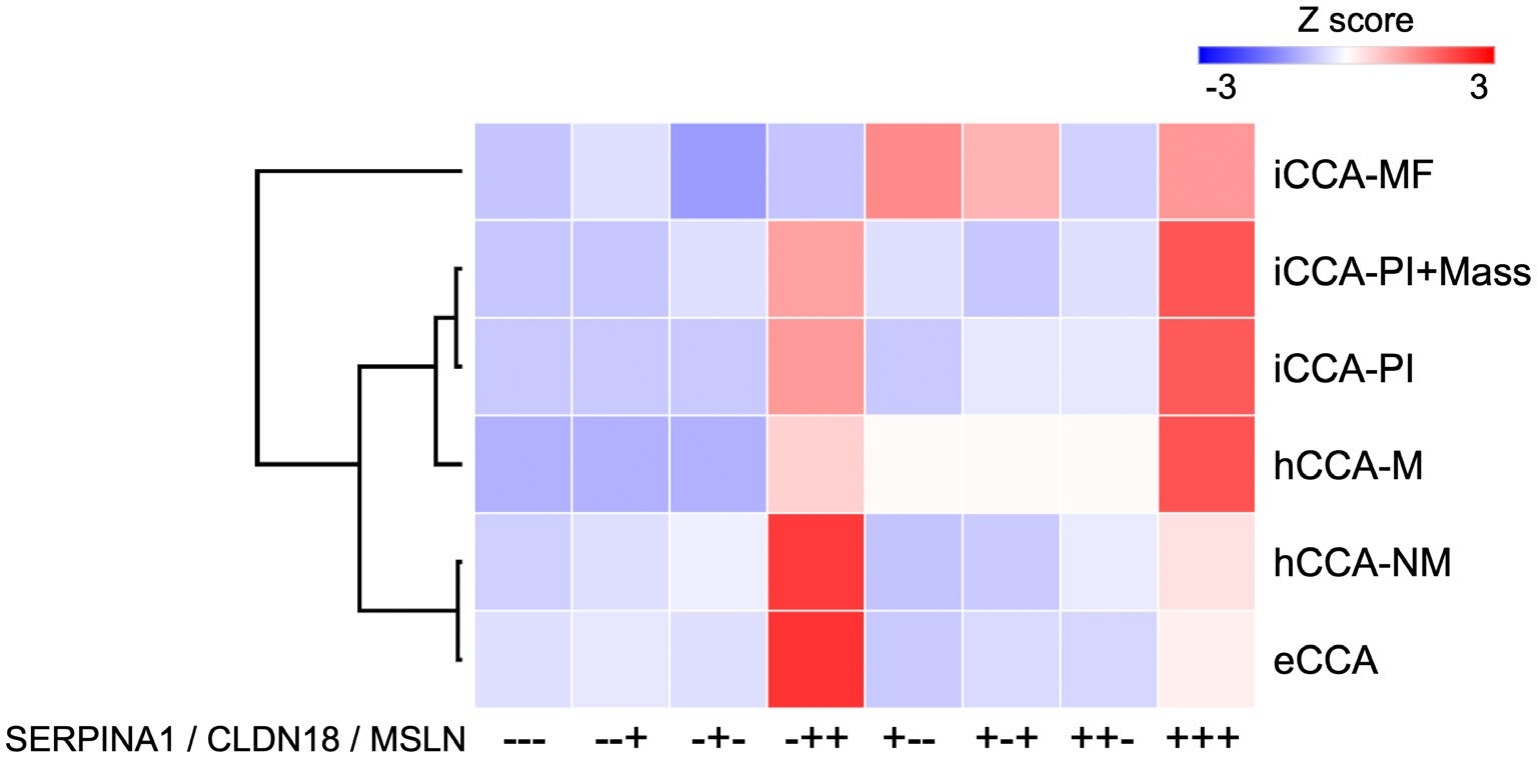
Expression patterns of the two extrahepatic‐context markers (CLDN18, MSLN) and the intrahepatic‐context marker (SERPINA1) in each tumor type in the validation set. The heat map was created according to the frequency of the expression patterns of the three contextual markers. hCCA‐NM and hCCA‐M were classified in different clusters; however, hCCA‐NM and eCCA were closely clustered. iCCA‐PI and iCCA‐PI+Mass were also closely related. iCCA‐MF formed a separate cluster distinct from the other types. hCCA‐M and iCCA‐PI (±Mass) constituted a cluster located between eCCA/hCCA‐NM and iCCA‐MF.

Hierarchical clustering of positive/negative patterns of the three contextual markers revealed three CCA clusters (Figure 5): eCCA and hCCA‐NM were closely related but were distinct from the cluster made up of hCCA‐M, iCCA‐PI, and iCCA‐PI+Mass. iCCA‐MF was different from the other subtypes.

### 3.6 Comparison of Liver Parenchymal Invasion of hCCA‐NM and hCCA‐M

**FIGURE 6:**
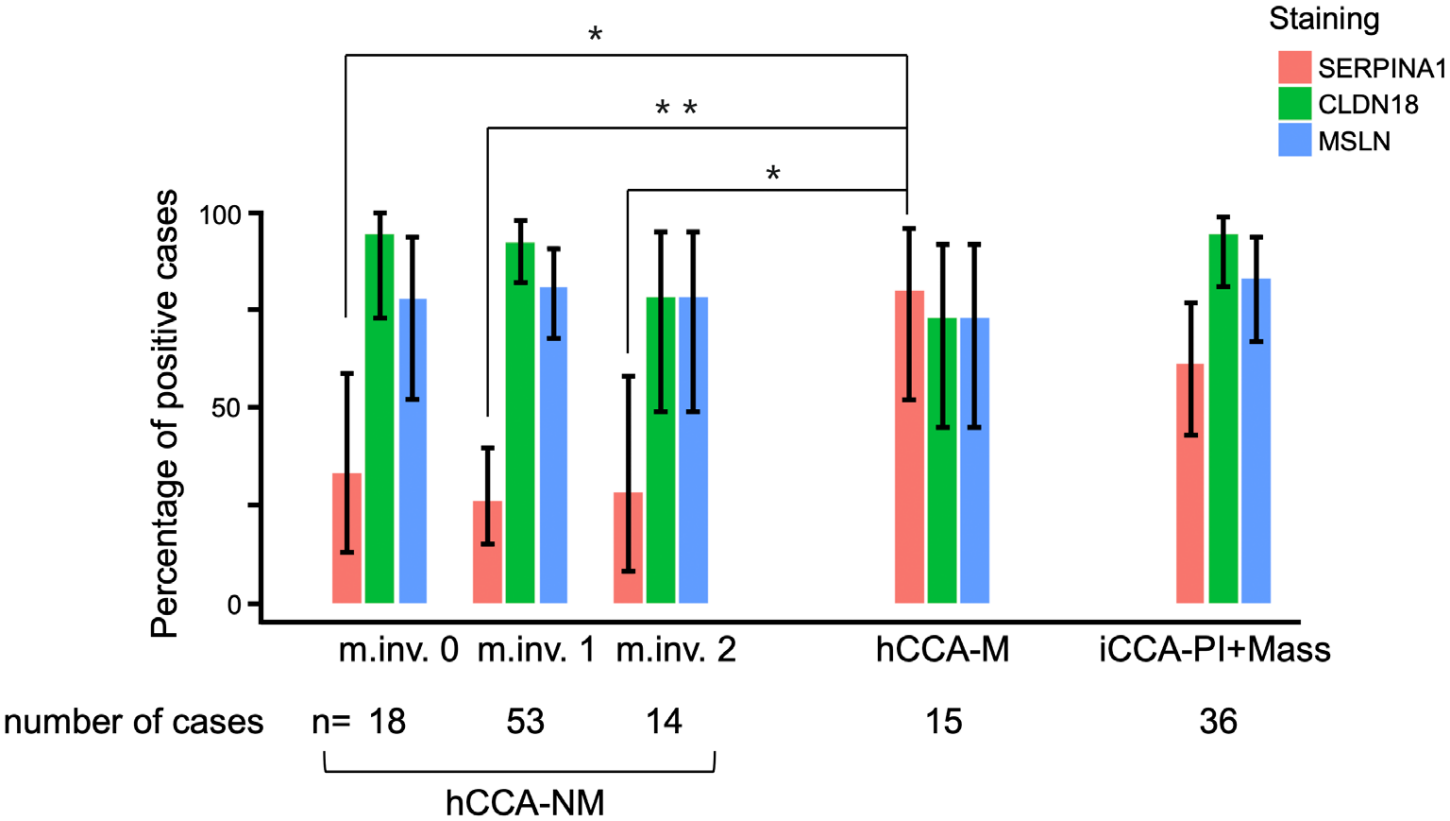
Marker protein expressions for different levels of microscopic liver parenchymal invasion of hCCA‐NM and for hCCA‐M. There were no significant differences in marker expression between microscopic liver parenchymal invasion levels 0, 1, and 2. hCCA‐M was significantly higher in SERPINA1 than hCCA‐NM with microscopic liver parenchymal invasion 0, 1, and 2; however, there were no significant differences for CLDN18 or MSLN. The percentage of positive cases was calculated based on the cut‐off values of each marker. Error bars show 95% confidence intervals. *Adjusted *p* < 0.05, **adjusted *p* < 0.01; m.inv., microscopic liver parenchymal invasion.

hCCA was evaluated with a focus on intrahepatic tumors (Figure 6). Similar expression levels of the three contextual marker proteins were observed in hCCA‐NM, irrespective of the degree of microscopic liver parenchymal invasion (m.inv. 0/1/2: SERPINA1 = 33%/26%/29%, CLDN18 = 94%/92%/79%, MSLN = 78%/81%/79%). Significantly lower expression of SERPINA1 was observed in hCCA‐NM than in hCCA‐M, irrespective of the presence or absence of microscopic parenchymal liver invasion (m.inv.0/1/2 vs. hCCA‐M = 33%/26%/29% vs. 80%, adjusted *p =* 0.042/*p* < 0.01/*p* = 0.028). There were no significant differences between hCCA‐M and iCCA‐PI/iCCA‐PI+Mass for any of the three contextual markers.

## 4 Discussion

In this study, we proposed a reliable methodology for identifying the primary site of phCCA near the hilar plate (which we defined as hCCA) with or without liver parenchymal tumor. Although pathological criteria for distinguishing these two tumor types were conceptually described in the Japanese clinical practice guidelines for intrahepatic cholangiocarcinoma (2022), [24] the supporting clinical datasets have not been systematically presented. In this study, we applied these diagnostic principles to a large cohort and incorporated molecular contextual analyses to further refine the pathological interpretation of hCCA.

To compare hCCA with the other tumor subtypes, we applied the diagnostic definition for differentiating iCCA from eCCA that we reported previously [7]. We initially examined the differences between intrahepatic and extrahepatic CCA using molecular pathology on samples of eCCA with a clearly defined extrahepatic primary and iCCA with a clearly defined intrahepatic primary. This approach allowed the identification of candidate protein markers that are differently expressed in eCCA and iCCA. These candidate proteins were used to establish an extrahepatic–intrahepatic reference contextual framework to position the two hCCA subtypes (i.e., hCCA‐M and hCCA‐NM, both termed phCCA in current guidelines) on this continuum, rather than to directly discriminate within hCCA.

In parallel, we examined the cholangiocarcinoma‐related markers listed in the WHO 2019 classification to assess how they aligned with this framework (Data S4). Among the 193 genes extracted in the analysis set, S100 (rank 31), MUC6 (rank 34), AGR2 (rank 38), and MUC1 (rank 75) are known large‐duct‐type markers that help subclassify iCCA. In our dataset, these genes showed high expression in eCCA, consistent with the biological overlap between large‐duct‐type iCCA and extrahepatic CCA. In contrast, CRP (rank 9), described as a small‐duct‐type feature, showed concordant transcript‐level enrichment; however, because no peer‐reviewed validation of the immunohistochemistry was available for eCCA or pancreas, CRP was not selected for downstream evaluation in this study. Although these WHO‐listed markers were not incorporated in the subsequent validation analysis, their expression patterns supported the overall framework of this study, in which hCCA subtypes were contextualized along an extrahepatic–intrahepatic continuum, rather than subclassified by ductal phenotype within iCCA.

SERPINA1, CLDN18, and MSLN were selected as candidate molecules. SERPINA1, also known as alpha1‐antitrypsin (A1AT), is a protease inhibitor belonging to the SERPIN superfamily [25]. SERPINA1 is typically expressed in liver [26] and is reportedly expressed in iCCA [17, 18, 27]. An association with the carcinogenesis of iCCA has also been reported [28]. SERPINA1 was selected as a representative contextual marker for iCCA. CLDN18 is a tight junction protein and regulates cell polarity [29]. A previous report examining CLDN18 in biliary tumors [30] suggested that CLDN18 is involved in the early carcinogenesis of CCAs derived from large bile ducts. This is based on the observation that CLDN18 is expressed in intraductal papillary neoplasms of the bile duct and biliary intraepithelial neoplasia, but not in normal bile duct and liver. Furthermore, that study highlighted the usefulness of CLDN18 in differentiating CCAs based on their expression patterns [30]. MSLN is a membrane glycoprotein that is originally expressed in mesothelial cells [31] but not in normal liver [23]. Although the precise function of MSLN remains unclear, its involvement in the early carcinogenesis of pancreatic cancer has been reported [32]. MSLN is reportedly highly expressed in eCCA, but less frequently expressed in iCCA [20, 21, 22], making it potentially useful in differentiating the CCA subtypes. Accordingly, CLDN18 and MSLN were selected as contextual markers of eCCA.

The primary site of hCCA‐NM and hCCA‐M can be estimated based on the findings of the current study and several previous studies. CLDN18 and MSLN are not expressed in large bile duct under normal circumstances, whereas they are induced at an early stage of carcinogenesis [20, 30]. These facts indicate that CLDN18 and MSLN proteins are highly expressed in so‐called large duct type CCAs, which are believed to originate from large bile duct cells. Because these two proteins were highly expressed in subtypes other than iCCA‐MF in the current study, these CCAs may originate from large bile ducts regardless of whether they are intra‐ or extrahepatic. In this context, these candidate proteins were used to establish an extrahepatic–intrahepatic reference contextual framework and to situate the two hCCA subtypes within it. CLDN18 and MSLN serve as indicators of extrahepatic context (which can overlap with large‐duct features) and were not intended to distinguish hCCA‐M from hCCA‐NM. However, consistent with this framework, we found a significant difference in SERPINA1 expression, depending on the primary site's location within or outside the liver. A public single‐cell dataset indicates that SERPINA1 is more highly expressed in intrahepatic bile duct cells than in extrahepatic bile duct cells (Data S5). SERPINA1 expression may indicate whether the primary site of CCA is extrahepatic or intrahepatic. The predominant SERPINA1 expression in intrahepatic primary tumors may be associated with the oxidation and carbonylation of A1AT, which reportedly contribute to the carcinogenic process and progression of CCA [17, 28]. The significant difference in SERPINA1 expression between hCCA‐NM and hCCA‐M indicates the necessity for distinguishing these two tumor types, in accordance with the presence or absence of grossly evident intrahepatic mass formation. Clustering analysis demonstrated that hCCA‐NM lacked intrahepatic contextual features and clustered together with eCCA, underscoring its extrahepatic pathological characteristics. Interestingly, hCCA‐M did not overlap with hCCA‐NM or eCCA, and hCCA‐M did not cluster with iCCA‐MF, which represents the small‐duct tumor type. Moreover, hCCA‐M exhibited expression patterns of CLDN18 and MSLN, which were identified as extrahepatic contextual markers and are commonly associated with tumors arising from large bile ducts. At the same time, hCCA‐M exhibited an intrahepatic contextual profile characterized by higher SERPINA1 expression. Therefore, based on our results, hCCA‐M showed proximity to large‐duct‐type tumors such as iCCA‐PI and iCCA‐PI+Mass, while representing a potentially distinct and independent cluster that is also distinct from iCCA‐MF. This finding provides support for our hypothesis that tumors with a gross intrahepatic mass should be classified as iCCA. In many cases, the gross intrahepatic mass of hCCA can be macroscopically identified, suggesting that the mass may be identified during the preoperative image review. Therefore, it may be possible to differentiate hCCA‐M and hCCA‐NM for unresected cases also.

One limitation of this study is that the number and combination of proteins evaluated were limited. We note that the three contextual markers applied here (CLDN18, MSLN, and SERPINA1) do not capture the full spectrum of possibilities; broader protein and genomic profiling—including direct comparative genomic analysis between hCCA‐M and hCCA‐NM—may further refine organ‐of‐origin inference. Further research is anticipated in the future.

In conclusion, the division of hCCA into hCCA‐NM and hCCA‐M revealed that these tumors have distinct molecular pathologies. The results show that invasion into the hilar region of iCCA and invasion into the liver parenchyma of eCCA can be distinguished by histological examination. These findings may contribute to refining the classification criteria of hCCA and aid in therapeutic decision‐making.

## Funding

This work was supported by Japan Society for the Promotion of Science (17K08769, 22K06985).

## Conflicts of Interest

The authors declare no conflicts of interest.

## Supporting information


**Data S1:** Summary of immunohistochemical staining of the three markers.

**Data S2:** Staining results for cases of eCCA and iCCA‐MF in the validation set.

**Data S3:** Representative immunohistochemical panels for hCCA‐M and hCCA‐NM.

**Data S4:** WHO‐related markers annotated on heatmap of eCCA vs. iCCA‐MF (analysis set).

**Data S5:** Single‐cell SERPINA1 gene expression of organoids developed from common bile duct cells/intrahepatic bile duct cells.

## Data Availability

The data that support the findings of this study are available from the corresponding author upon reasonable request.
