# Supplementary material for: Gross Intrahepatic Mass Formation Predicts the Primary Site of Perihilar Cholangiocarcinoma Based on Molecular Pathologic Studies
Source: J Hepatobiliary Pancreat Sci. 2026 Feb 1;33(4):284–93. doi: 10.1002/jhbp.70077 (PMC13113198; doi:10.1002/jhbp.70077)
Supplement: Supplementary file 1 — Data S1: Summary of immunohistochemical staining of the three markers. [file JHBP-33-284-s002.docx]

Supplemental data 1 Summary of immunohistochemical staining of three markers

| Gene | Antigen | Product | Host | Dilution | Details of pre-incubation | Expression site diagnosed as positive | Internal positive control |
| --- | --- | --- | --- | --- | --- | --- | --- |
| SERPINA1 | Alpha 1 antitrypsin | ab9373, Abcam, Cambridge, UK | Rabbit  Polyclonal | 1 : 600 | Heated at 121°C in 0.01 mol ⁄ l sodium citrate buffer, pH 7.0, for 10 min | Tumor cells with cytoplasmic expression equal to or more than that of the internal positive control | Liver (if no liver: interstitial fiber） |
| CLDN18 | Claudin 18 | 38-8000, Thermo Fisher Scientific, Waltham, MA, USA | Rabbit  Polyclonal | 1 : 250 | Heated at 121°C in 0.01 mol ⁄ l sodium citrate buffer, pH 7.0, for 10 min | Tumor cells with cellular membrane expression | None |
| MSLN | Mesothelin | ab93620, Abcam, Cambridge, UK | Rabbit  Monoclonal | 1 : 1000 | Heated at 97°C in 0.01 mol ⁄ l sodium citrate buffer, pH 7.0, for 20 min | Tumor cells with cytoplasm or cellular membrane expression | None |
