# Supplementary figures and images for: Gross Intrahepatic Mass Formation Predicts the Primary Site of Perihilar Cholangiocarcinoma Based on Molecular Pathologic Studies
Source: J Hepatobiliary Pancreat Sci. 2026 Feb 1;33(4):284–93. doi: 10.1002/jhbp.70077 (PMC13113198; doi:10.1002/jhbp.70077)

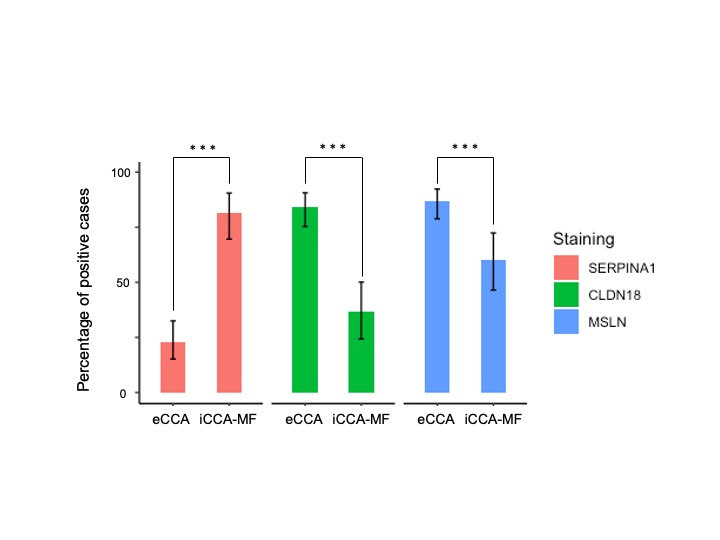

Supplement: Supplementary file 2 — Data S2: Staining results for cases of eCCA and iCCA‐MF in the validation set. [file JHBP-33-284-s004.jpeg]

■ iCCA-MF (n = 39)

■ eCCA (n = 9)

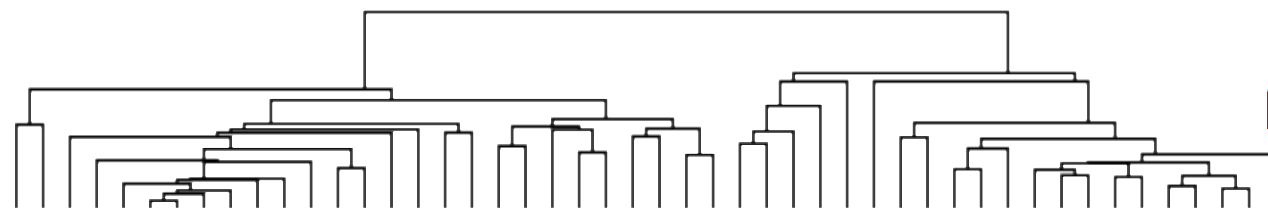

S100  
AGR2 *CLDN18*

— *MUC1*

— *MSLN*

— *MUC6*

— *SERPINA1*

— *CRP*

Gene expression  
Z score

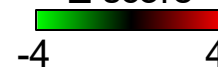

group A

group B

Supplement: Supplementary file 4 — Data S4: WHO‐related markers annotated on heatmap of eCCA vs. iCCA‐MF (analysis set). [file JHBP-33-284-s003.pdf]

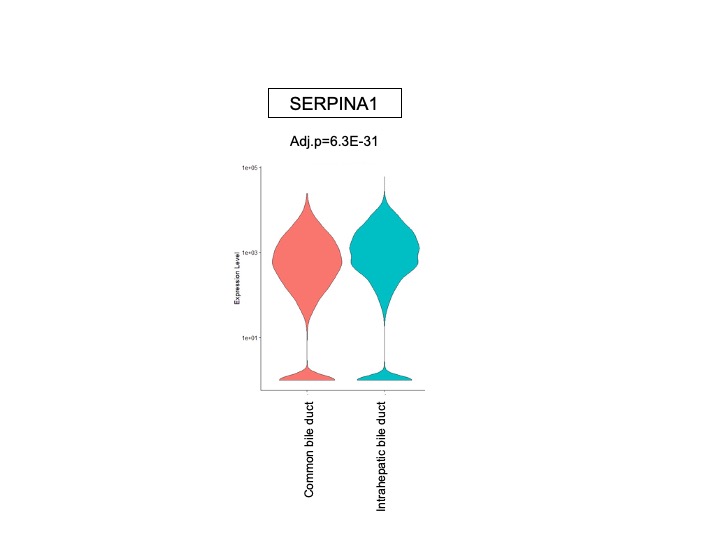

Supplement: Supplementary file 5 — Data S5: Single‐cell SERPINA1 gene expression of organoids developed from common bile duct cells/intrahepatic bile duct cells. [file JHBP-33-284-s005.jpeg]
