# Supplementary material for: Gross Intrahepatic Mass Formation Predicts the Primary Site of Perihilar Cholangiocarcinoma Based on Molecular Pathologic Studies
Source: J Hepatobiliary Pancreat Sci. 2026 Feb 1;33(4):284–93. doi: 10.1002/jhbp.70077 (PMC13113198; doi:10.1002/jhbp.70077)
Supplement: Supplementary file 3 — Data S3: Representative immunohistochemical panels for hCCA‐M and hCCA‐NM. [file JHBP-33-284-s001.pdf]

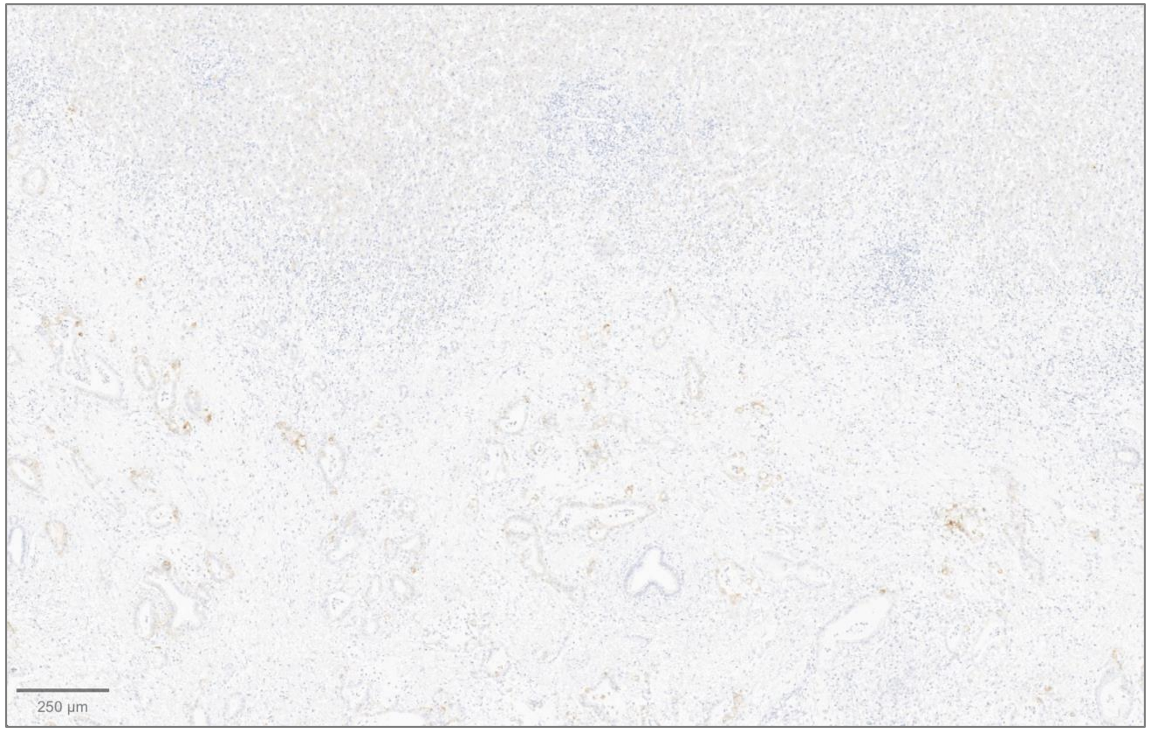


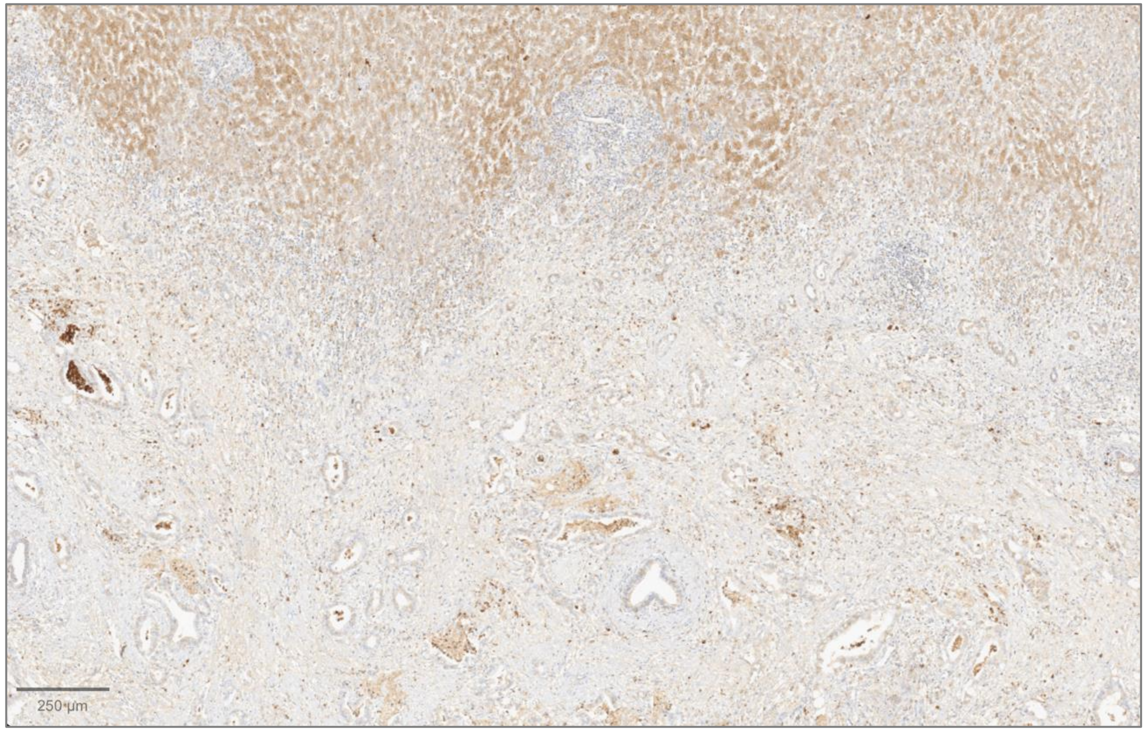


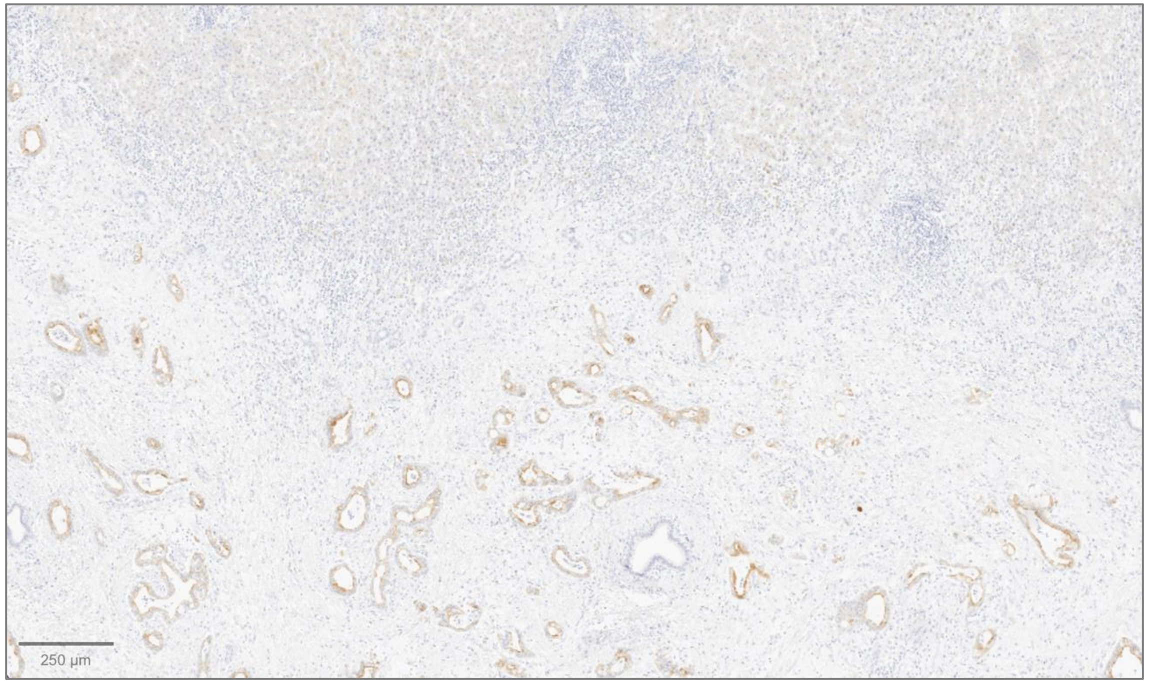


Scale bar = 250μm

hCCA-NM

| CLDN18 | SERPINA1 |
| --- | --- |
| MSLN | |

※In this representative hCCA-NM case, focal SERPINA1-positive tumor cells were observed; however, because the proportion of positive tumor cells was ≤33%, the case was classified as SERPINA1-negative.

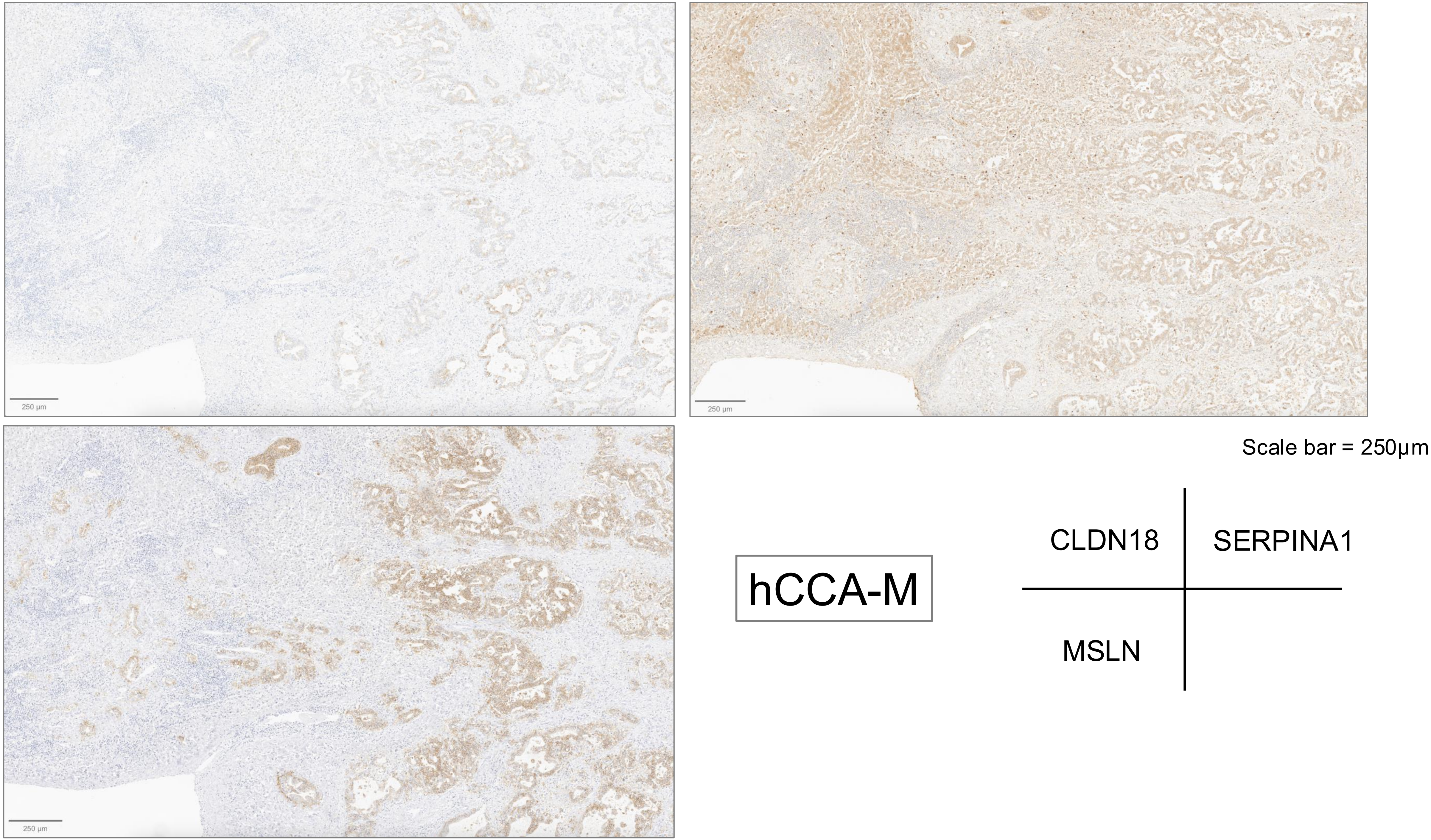
250 µm
250 µm
250 µm
Scale bar = 250µm
hCCA-M
CLDN18
SERPINA1
MSLN
